# Supplementary material for: Prevalence and Characterization of ESBL/AmpC Producing Escherichia coli from Fresh Meat in Portugal
Source: Antibiotics (Basel). 2021 Nov 1;10(11):1333. doi: 10.3390/antibiotics10111333 (PMC8615096; doi:10.3390/antibiotics10111333)
Supplement: Supplementary file 1 [file antibiotics-10-01333-s001.zip › antibiotics-1436525-supplementary.pdf]

Supplementary Materials

Table S1. Occurrence of MDR phenotypes in beef (*n* = 26), pork (*n* = 23) and broiler meat (*n* = 60)

**Multidrug Resistance Patterns**	**Beef**		**Pork**		**Broiler meat**		**Total**	
	**Nº of isolates**	**(P)**	**Nº of isolates**	**(P)**	**Nº of isolates**	**(P)**	**Nº of isolates**	**(P)**
AMP-FOT-TAZ-FEP-CIP-SMX	0	0	0	0	1	1.8	1	1.0
**AMP-FOT-TAZ-FEP-TET-SMX**	3	13.6	4	20.0	0	0	7	7.0
AMP-FOT-TAZ-FEP-NAL-CIP-TET	0	0	0	0	1	1.8	1	1.0
AMP-FOT-TAZ-NAL-CIP-SMX-TMP	0	0	0	0	1	1.8	1	1.0
AMP-FOT-TAZ-FEP-FOX-TET-TMP	1	4.5	0	0	0	0	1	1.0
AMP-FOT-TAZ-FEP-NAL-SMX-TMP	0	0	0	0	1	1.8	1	1.0
**AMP-FOT-TAZ-FEP-TET-SMX-TMP**	0	0	2	10.0	1	1.8	3	3.0
AMP-FOT-FEP-NAL-CIP-SMX-TMP	1	4.5	0	0	1	1.8	2	2.0
AMP-FOT-TAZ-FEP-TET-SMX-CHL	0	0	0	0	2	3.5	2	2.0
AMP-FOT-FEP-TET-SMX-TMP-CHL	0	0	1	5.0	0	0	1	1.0
AMP-FOT-TAZ-FEP-TET-SMX-GEN	0	0	1	5.0	0	0	1	1.0
AMP-FOT-TAZ-NAL-CIP-TET-TMP-CHL	0	0	1	5.0	0	0	1	1.0
AMP-FOT-TAZ-NAL-CIP-TET-SMX-TMP	1	4.5	0	0	0	0	1	1.0
AMP-FOT-TAZ-FEP-NAL-CIP-TET-TMP	1	4.5	0	0	0	0	1	1.0
AMP-FOT-TAZ-FEP-FOX-NAL-CIP-TET	1	4.5	0	0	0	0	1	1.0
**AMP-FOT-TAZ-FEP-NAL-CIP-TET-SMX**	1	4.5	1	5.0	5	8.8	7	7.0
AMP-FOT-TAZ-FEP-NAL-CIP-SMX-CHL	1	4.5	0	0	0	0	1	1.0
AMP-FOT-TAZ-FEP-NAL-CIP-SMX-TMP	0	0	0	0	1	1.8	1	1.0
AMP-FOT-TAZ-FEP-FOX-NAL-TET-GEN	0	0	1	5.0	0	0	1	1.0
AMP-FOT-FEP-NAL-CIP-TET-SMX-TMP	0	0	0	0	1	1.8	1	1.0
AMP-FOT-FEP-NAL-CIP-TET-SMX-CHL	1	4.5	0	0	0	0	1	1.0

Multidrug Resistance Patterns	Beef		Pork		Broiler meat		Total	
	**Nº of isolates**	**(P)**	**Nº of isolates**	**(P)**	**Nº of isolates**	**(P)**	**Nº of isolates**	**(P)**
AMP-FOT-FEP-CIP-TET-SMX-TMP-CHL	0	0	1	5.0	0	0	1	1.0
AMP-FOT-TAZ-FEP-TET-SMX-TMP-CHL	1	4.5	0	0	0	0	1	1.0
**AMP-FOT-TAZ-FEP-NAL-CIP-TET-SMX-CHL**	1	4.5	0	0	4	7.0	5	5.0
AMP-FOT-TAZ-NAL-CIP-COL-TET-SMX-CHL	0	0	0	0	1	1.8	1	1.0
AMP-FOT-TAZ-NAL-CIP-TET-SMX-TMP-CHL	0	0	0	0	1	1.8	1	1.0
AMP-FOT-TAZ-NAL-CIP-TET-SMX-TMP-GEN	0	0	0	0	1	1.8	1	1.0
AMP-FOT-TAZ-FEP-FOX-ETP-NAL-CIP-TMP	0	0	1	5.0	0	0	1	1.0
AMP-FOT-TAZ-FEP-FOX-CIP-TET-SMX-TMP	0	0	1	5.0	0	0	1	1.0
**AMP-FOT-TAZ-FEP-NAL-CIP-TET-SMX-TMP**	0	0	3	15.0	8	14.0	11	11.0
**AMP-FOT-TAZ-FEP-NAL-CIP-SMX-TMP-CHL**	2	9.1	0	0	0	0	2	2.0
AMP-FOT-TAZ-FEP-NAL-TET-SMX-TMP-CHL	0	0	0	0	1	1.8	1	1.0
**AMP-FOT-TAZ-FOX-NAL-CIP-TET-SMX-TMP**	0	0	1	5.0	5	8.8	6	6.0
AMP-FOT-TAZ-FOX-NAL-CIP-TET-SMX-TMP-CHL	0	0	0	0	2	3.5	2	2.0
AMP-FOT-TAZ-FOX-NAL-CIP-TET-SMX-TMP-GEN	0	0	0	0	1	1.8	1	1.0
**AMP-FOT-TAZ-FEP-NAL-CIP-TET-**	2	9.1	0	0	7	12.3	9	9.0

**SMX-TMP-CHL**								
AMP-FOT-TAZ-FEP-FOX-NAL-CIP-TET-SMX-TMP	0	0	0	0	3	5.3	3	3.0
AMP-FOT-TAZ-FEP-NAL-CIP-COL-TET-SMX-TMP	0	0	0	0	1	1.8	1	1.0
AMP-FOT-TAZ-FEP-FOX-ETP-NAL-CIP-SMX-TMP	1	4.5	0	0	0	0	1	1.0
AMP-FOT-TAZ-FEP-FOX-NAL-CIP-TET-SMX-GEN	0	0	0	0	1	1.8	1	1.0
AMP-FOT-TAZ-FEP-NAL-CIP-TET-SMX-TMP-GEN	0	0	0	0	2	3.5	2	2.0
AMP-FOT-TAZ-FEP-FOX-NAL-CIP-TET-SMX-TMP-CHL	0	0	1	5.0	0	0	1	1.0
**AMP-FOT-TAZ-FEP-NAL-CIP-COL-TET-SMX-TMP-CHL**	2	9.1	1	5.0	1	1.8	4	4.0
**AMP-FOT-TAZ-FEP-NAL-CIP-TET-SMX-TMP-GEN-CHL**	2	9.1	0	0	3	5.3	5	5.0

SMX, Sulfamethoxazole; **TMP**, Trimethoprim; **CIP**, Ciprofloxacine; **TET**, Tetracycline; **NAL**, Nalidixic Acid; **CLO**, Chloramphenicol; **COL**, colistine; **AMP**, Ampicillin; **GEN**, Gentamicine; **FOT**, Cefotaxime; **TAZ**, Ceftazidime; **FOX**, Cefoxitin; **FEP**, Cefepime;**P**, prevalence; Highlighted in **bold** are the most frequent MDR phenotypes
